# Interoception and Social Connection

**DOI:** 10.3389/fpsyg.2019.02589

**Published:** 2019-11-26

**Authors:** Andrew J. Arnold, Piotr Winkielman, Karen Dobkins

**Affiliations:** ^1^Department of Psychology, University of California, San Diego, San Diego, CA, United States; ^2^Department of Psychology, Knox College, Galesburg, IL, United States; ^3^Department of Psychology, SWPS University of Social Sciences and Humanities, Warsaw, Poland

**Keywords:** interoception, health, emotion, loneliness, social cognition, social connection

## Abstract

Interoception – the process of sensing bodily signals – has gained much interest in recent years, due to its role in physical and mental well-being. Here, we focus on the role of interoception in *social connection*, which is a relatively new and growing research area. Studies in this area suggest that interoception may help in appraising physiological signals in social situations, but also that (challenging) social situations may reduce interoceptive processing by shifting attention from internally- to externally- focused. We discuss potential mechanisms for the influence of interoception on social connection and highlight that flexibility in engaging interoception in social situations may be particularly important. We end with a discussion of loneliness – an extreme case of poor social connection, which is associated with physiological decline and increased mortality risk, and propose that interoceptive dysregulation is involved. We suggest that interventions aimed to improve interoceptive abilities, such as mindfulness-based meditation practices, may be key for alleviating loneliness and improving social connection.

## Introduction

Interoception – the process of sensing bodily signals – holds importance for physiological functioning (Craig, 2015). In recent years, there has been a surge of interest in interoception because of its impact on physical health (Quadt et al., 2018), mental health (Khalsa et al., 2018), as well as emotional functioning in general (Critchley and Garfinkel, 2017). Here, we focus on a growing literature investigating the link between interoception and *social connection.* This represents an important focus within the broader area of how interoception influences many types of social phenomena, or “social interoception.” We review some illustrative studies and discuss some potential mechanisms of this link. We then end with a discussion about why understanding the role of interoception in social connection is important for physical and mental health, with a focus on loneliness.

### Social Interoception

Before turning to the role of interoception in social connection, we define interoception as the sensing of internal physiological states of the body (e.g., hunger, micturition, thirst, temperature), which serves as a means of regulating and maintaining homeostatic conditions (Craig, 2009; Sterling, 2011). Interoception involves the central processing of bottom-up, afferent signals from the body along with top-down regulatory directives. These physiological signals may (or may not) be represented as subjective feelings, and then may lead to behaviors to adjust the current state. Interoception is functionally distinct from exteroceptive senses (e.g., vision, audition), as well as proprioception (i.e., sensing the position of muscles/joints), although it interacts with these senses through multimodal sensory integration (Craig, 2015). A commonplace example of interoception is sensing a full bladder. In a healthy human, this results in a subjective feeling, with the behavioral adjustment being the action of emptying the bladder (though note that in some cases such behavior may bypass conscious feeling and volitional action; Griffiths, 2015).

But how does interception function in *social* situations? Take the example of a threatening social interaction. Here, the bodily response might entail an increase in heart rate – a reaction that serves to enhance alertness (e.g., preparing one to either argue one’s case or walk away). These physiological changes may (or may not) be felt and they may (or may not) generate affect, as we elaborate below. Interestingly, there is also a prediction component to this process and the role of “predictive interoceptive coding” is gaining understanding, but outside the scope of this review (see Barrett et al., 2016).

### Dimensions of Interoception

Before reviewing studies that have investigated the relationship between interoception and social connection, we describe dimensions of interoception and ways to measure it in the laboratory. This is to highlight the multidimensionality of the construct. Garfinkel et al. (2015) define three key dimensions. Two are more objective – interoceptive accuracy (IAcc) and interoceptive awareness (IAw). One is more subjective – interoceptive sensibility (IS). Each of these are separate dimensions and do not necessarily correlate with one another (e.g., see Garfinkel et al., 2016).

Interoceptive accuracy refers to an individual’s ability to shift attention internally and accurately track their physiological state. The most commonly used task involves individuals monitoring their heartbeat by asking them to either count their number of heartbeats during variable time intervals, referred to as *heartbeat counting* (HBC^1^), or determine whether an external signal is synchronous with their own heartbeat, referred to as *heartbeat discrimination*. Note that unless stated otherwise, the IAcc studies discussed in this review used HBC.

Interoceptive awareness is a metacognitive construct. It is assessed by conducting the IAcc task, as well as asking individuals to rate their confidence in their guesses, collecting data across many trials, and testing the correlation between trial-by-trial IAcc scores and confidence ratings. A strong correlation indicates one has good metacognitive awareness of interoceptive accuracy.

Interoceptive sensibility is one’s subjective tendency to perceive, appraise, and use physiological signals, as measured by self-report questionnaires. One such questionnaire is the MAIA (Mehling et al., 2012) which has eight subscales, including: noticing, not-distracting, not-worrying, attention regulation, emotional awareness, self-regulation, body listening, and body trusting. These subscales index different attentional and appraisal “styles” with regard to processing and using physiological signals. Research shows that going beyond IAcc and IAw and distinguishing various attentional and regulatory aspects of interoception is important for understanding its role in typical social processing and psychopathological conditions (Mehling, 2016).

### The Role of Interoception in Social Connection

There are several studies suggesting an intriguing link between interoception and social connection. The first type of research is correlational in nature, showing that trait interoceptive ability (IAcc) is associated with social reactivity. One study used the Trier social stress test to induce negative affect through impromptu public speaking (Werner et al., 2009), while another study subjected participants to social exclusion in a conversation with confederates, which is also presumably stressful (Werner et al., 2013). In both studies, people higher in IAcc reported less negative affect after the challenging social situation, despite their physiological reactivity being comparable to participants with lower IAcc. Such results are consistent with the idea that it is not the physiological response *per se* that causes social stress (in the form of negative affect), but one’s subjective appraisal of it (Kudielka et al., 2007). This leads to the interesting idea that perhaps greater IAcc allows one to identify the physiological response as resulting from an objective, external “social situation” rather than an attribute of oneself. This could reflect better emotional regulation in social situations (Werner et al., 2013). Finally, another study induced social exclusion with the Cyberball game and similarly found that higher IAcc was associated with less negative affect as well as behavioral affiliation tendency, as measured by preferred interpersonal distance (Pollatos et al., 2015). Recently, conflicting (null) results on the effect of IAcc buffering against negative affect from Cyberball were found – although these researchers used different measures for independent and dependent variables for affect, this may underscore the (contextual) complexity of interoceptive contributions to emotional resilience (Zamariola et al., 2019).

Although not directly manipulating social interaction, another intriguing study showed how trait interoceptive accuracy (IAcc) relates to peripersonal space – the region just surrounding one’s body in which multisensory integration is heightened for salient processing (Ferri et al., 2013). This study showed that the higher IAcc is associated with greater autonomic reactivity in response to seeing another person’s hand entering their peripersonal space (i.e., 20 cm from their own hand). Critically, this result was not seen when the entering object is a metal stick. Given that an approaching human hand is a proxy for social connection, this result is consistent with the possibility that interoception facilitates noticing of a potential social connection. Interestingly, social context has also been reported to shape the mapping of peripersonal space – becoming more expansive and inclusive when participants interact with someone perceived to be cooperative or moral (Teneggi et al., 2013; Ardizzi and Ferri, 2018; Pellencin et al., 2018). These results open the door for the use of peripersonal space tasks as a way to measure the degree of social connection in future studies, an idea we return to below.

The second type of research addressing the link between interoception and social connection (or lack thereof) is experimental in nature, showing that social situations can elicit acute changes in interoceptive ability. One study found that social exclusion (manipulated by the Cyberball game) caused an acute decrease in IAcc, compared to baseline levels (Durlik and Tsakiris, 2015). These results can be explained by a “shifting” of attention from interoceptive to exteroceptive processing, increasing social attention as a means to re-affiliate after being excluded (Powers and Heatherton, 2012), which we expand upon below. It is important to note that a decrease in IAcc in response to social exclusion is not necessarily contradictory to findings showing that reaction to social exclusion is less negative in people with higher trait IAcc (described above). After all, it is entirely possible that both are true: (i) better IAcc skills allow one to properly appraise one’s internal state in response to social exclusion, *and* (ii) social exclusion leads to an overall reduction in IAcc due to a switch from internal to external (social) attention. These considerations bring us to a broader discussion of mechanisms linking interoception and social connection, below.

### Mechanisms for Linking Interoception With Social Connection: Enhanced Emotional Discernment and Attentional Switching

Given that there are links between interoception and social processing, what might be the underlying mechanisms? One idea, which we refer to as the “*enhanced emotional discernment hypothesis*,” is that better ability to feel bodily reactions translates to having a richer emotional experience. In turn, richer emotional experience may facilitate greater understanding of others’ emotions, and empathy – the process of understanding, sharing, and/or responding to others’ emotions (Decety, 2011; Zaki, 2014). Such empathic understanding and emotion sharing should then facilitate social connection. There is some evidence that is consistent with this hypothesis. First, with respect to whether heightened interoception is associated with rich emotional experience, it has been reported that people higher in IAcc tend to feel emotions more intensely (Barrett et al., 2004) and use emotion regulation strategies of reappraisal and suppression more frequently (Kever et al., 2015). In addition, interoceptive dysregulation appears to underlie alexithymia – difficulty identifying one’s own emotions and sensations (Brewer et al., 2016). Alexithymia is a common hallmark of psychological disorders such as Autism Spectrum Conditions that present with social difficulties (Bird and Cook, 2013). With respect to the connection between understanding one’s own emotions and those of others, a meta-analysis has shown that alexithymia predicts difficulties in recognizing emotions in others (Grynberg et al., 2012).

Two other studies more directly tie interoceptive abilities with feeling emotions of others. Grynberg and Pollatos (2015) found that IAcc is positively correlated with the degree to which one reports experiencing cognitive and affective empathy. In a similar vein, Terasawa et al. (2014) found that IAcc is positively associated with the degree of emotional contagion participants experience when identifying and rating faces expressing joy and sadness, but not anger and disgust. Interestingly, joy and sadness seem to provide the greatest potential for social understanding and connection (Hess and Fischer, 2013). Consistently, spontaneous mimicry of other people’s expressions of joy is reduced in people who are socially disconnected – i.e., lonely (Arnold and Winkielman, unpublished). This result, in contrast to the last, may suggest interoceptive dysregulation in loneliness – a topic we explore below. In short, such findings are consistent with the possibility that interoceptive ability facilitates social connection. In fact, one can imagine a positive feedback loop – once one’s social life starts to “feel right,” one can start to trust/rely on their interoceptive signals even more in order to learn from social affective experience.

A second possible mechanism, which we refer to as the “attentional switching hypothesis,” supposes that if one’s attention is drawn toward external events (including social situations that are challenging), this may reduce attentional resources for monitoring internal states (i.e., interoception). A few lines of evidence support this view. As described above, studies have shown that social exclusion causes reduction in interoceptive abilities (IAcc). Another study demonstrated that repeated exposure to angry faces leads to a decrease on a neural measure of interoceptive processing – the heartbeat-evoked brain potential (Pollatos and Schandry, 2004), and also an increase in visually evoked potential to the angry faces (Marshall et al., 2018). Given that the heartbeat-evoked potential indexes interoceptive processing, these data provide more direct evidence for the switching hypothesis – i.e., challenging social situations lead to an acute switch from interoceptive to exteroceptive attention. Another relevant study showed that administering oxytocin, which is implicated in social salience (Shamay-Tsoory and Abu-Akel, 2016), decreases IAcc in participants when they are being presented with emotional faces, but not in a control condition when they are exposed to a blank screen (Yao et al., 2018). Given that oxytocin increases social attention, these results are also consistent with exteroceptive social attention drawing resources away from interoceptive focus.

**FIGURE 1:**
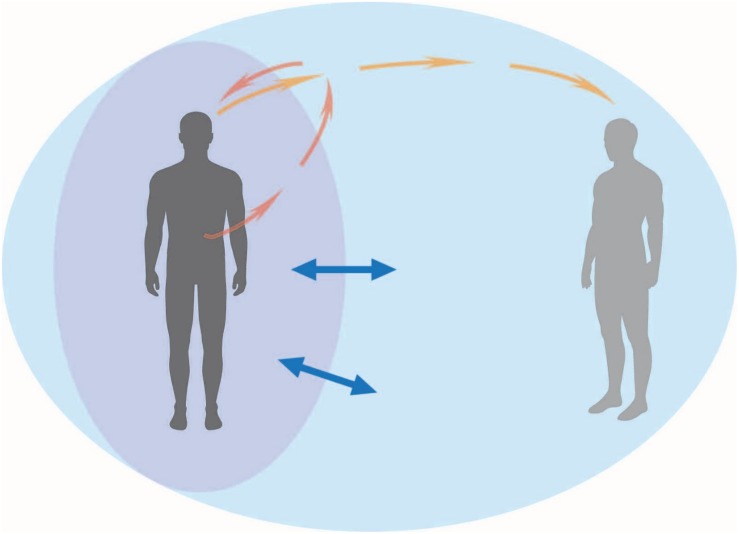
An interoceptive human in a social situation. In this diagram, the arrows represent attentional focus for the social actor on the left: dark orange for interoceptive attention, light orange for exteroceptive attention. Dynamic interplay between these two modalities represent an attentional tradeoff that can be triggered by certain social situations – social attention (too) outwardly engaged may detract from moment-by-moment interoceptive focus. The violet oval represents peripersonal space, which may interact with interoceptive processing in social situations. The blue double-sided arrows represent the tendency for peripersonal space mapping to expand or contract, based on social context. The large light blue oval represents the overall social environment.

Since the switching hypothesis suggests a tradeoff between internal and external attention, it is important to consider which may best serve social connection. We propose that the key to interoception facilitating social connection is the ability to *flexibly* shift between interoceptive (emotional signals) attention and exteroceptive (social) attention. For example, when encountering a stranger or developing a friendship, it is important to be able to adaptively (and quickly) read the emotions and intentions of another person, as well as one’s own reactions to them. Interestingly, Ainley et al. (2016) recently proposed that IAcc scores reflect the ability to adaptively weight interoceptive signals over others competing for attention, which is in line with our account. One possible way to test the effectiveness of interoceptive vs. exteroceptive attention might involve directly instructing participants to shift from one to the other, and seeing how that affects social connection (measured with peripersonal space, for example). Interacting attentional modalities, as well as attentional field fluctuations within peripersonal space, that are potentially involved in a social situation are depicted in Figure 1. Note that these attentional investments *can* be dynamic and interactive.

### Social Connection in Mental and Physical Health: Potential for Interoception Interventions

Social connection and belonging motivate much of human behavior and cognition (Baumeister and Leary, 1995). Social connection is required for health, with some of the best evidence coming from studies on loneliness – perceived social isolation – which results when one’s social relationships do not fulfill one’s desired level of social connectedness (Cacioppo and Patrick, 2008). The feeling of loneliness, thus, is subjective and distinct from objective measures of social isolation. Given the strong human need for social connection, it is not surprising that loneliness is associated with physiological decline and increased mortality risk (Luo et al., 2012). In longitudinal studies that track the onset of negative psychological states, loneliness has been shown to predict increases in depression, but not vice versa (Cacioppo et al., 2010), and shows a reciprocal relationship with subjective well-being (VanderWeele et al., 2012). Loneliness is also associated with low self-reported empathy (Beadle et al., 2012), which could imply interoceptive dysregulation. Given the negative health consequences of poor social connectedness, it is important to consider ways to alleviate (increasing) loneliness in society (Will, 2018).

With regard to the above “attentional switching hypothesis,” could loneliness involve heightened exteroceptive attention, and reduced interoceptive attention? Interestingly, Cacioppo and Hawkley (2009) proposed an (exteroceptive) attentional bias as key to maintaining loneliness and making it hard to overcome: *hypervigilance for social threat* (Cacioppo and Hawkley, 2009). This implicit attentional bias focuses on negative over positive social stimuli, and may coexist with altered interoceptive attention. We speculate that in chronic loneliness, the maladaptive attentional focus that shifts processing away from positive social signals to external negative social signals, could also reduce the degree and flexibility of interoceptive processing.

In order to facilitate social sensitivity and reduce loneliness, we suggest that improving one’s interoceptive abilities may be key. This idea also follows from the “enhanced emotional discernment hypothesis,” as outline above. Being able to accurately “tune in” to one’s own internal (emotional) states and properly used them in social judgments may improve social connection and decrease loneliness. In fact, data from our lab show a strong negative correlation between IS (as assessed with the MAIA scales) and loneliness (as assessed with the UCLA questionnaire). Interestingly, the subscale that most strongly predicts loneliness is “body trusting,” suggesting that trust (appraisal of acceptance) in one’s bodily signals may help “buffer” one from loneliness (Arnold and Dobkins, unpublished). As mentioned, trust and proper use of bodily signals could facilitate adaptive learning from social and affective experiences.

If good interoception does buffer against loneliness, interventions to reduce loneliness should focus on improving interoception. While loneliness reduction interventions remain difficult (Cacioppo et al., 2015), some of the most effective interventions have used mindfulness-based stress reduction (MBSR) techniques (Creswell et al., 2012; Lindsay et al., 2019). Similar meditation techniques have been shown to improve both IS (measured with MAIA; Bornemann et al., 2015) and interoceptive accuracy (Bornemann and Singer, 2017). This suggests that the effectiveness of MBSR in reducing loneliness may be mediated in part by improvements in interoception. Of course, mindfulness involves multiple cognitive and affective processes and influences many different aspects of subjective and social experience (Singer and Engert, 2019). Plainly, more research is needed to identify specific mechanisms associated with loneliness and poor interoception that could be targeted in further interventions for psychosocial function. Nevertheless, it appears that the growing field of “social interoception” represents a fruitful area of research for better understanding and treating not only loneliness, but other psychopathological conditions, as well as improving social health for all.

## Author Contributions

AA conceived the study and drafted the manuscript, which was written with KD and PW. All authors agreed to the current form of the manuscript.

## Conflict of Interest

The authors declare that the research was conducted in the absence of any commercial or financial relationships that could be construed as a potential conflict of interest.

## Footnotes

1 Recently, some researchers expressed concerns about HBC as a measure of interoceptive accuracy because knowledge of one’s own resting heart rate may confound HBC performance (Ring et al., 2015). Specifically, participants tend to underestimate their heartbeat counts and re-test reliability is not high (Zamariola et al., 2018). We appreciate this point but highlight other qualitative dimensions of interoception as relevant to social cognition, in addition to citing papers that successfully used HBC as a measure of interoceptive accuracy.
